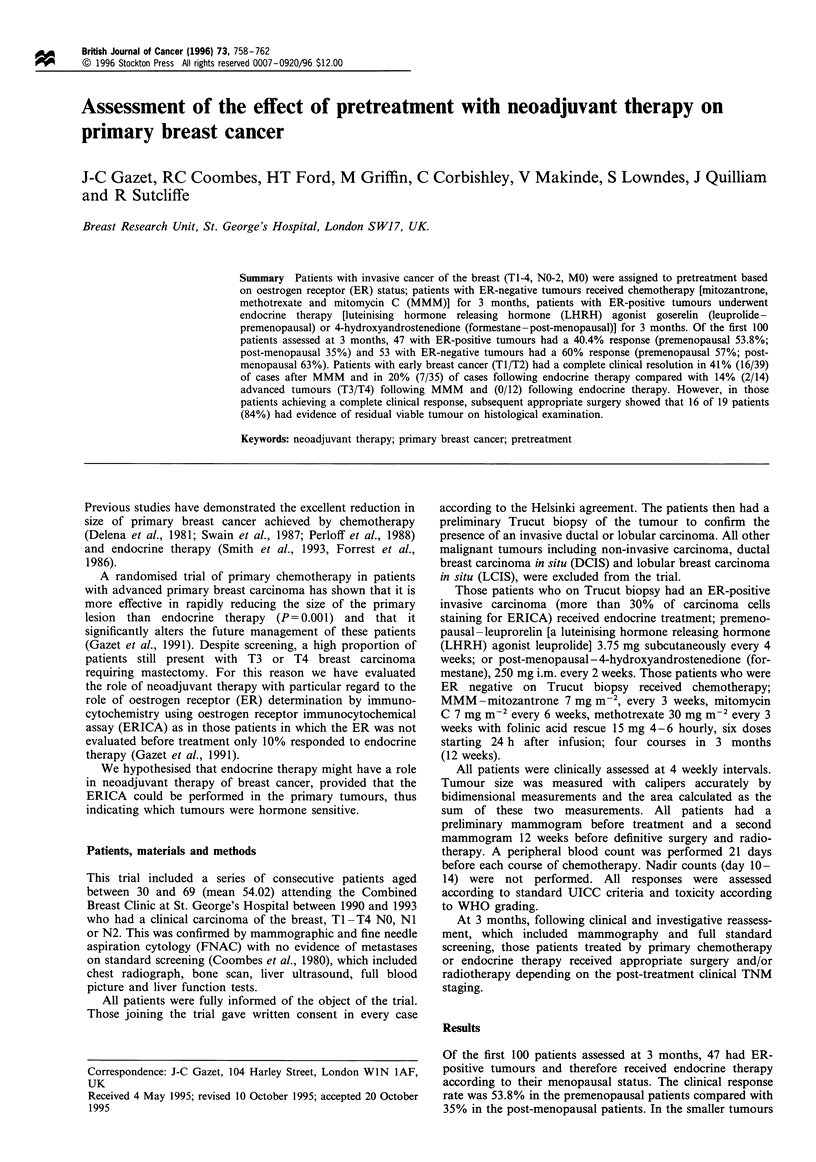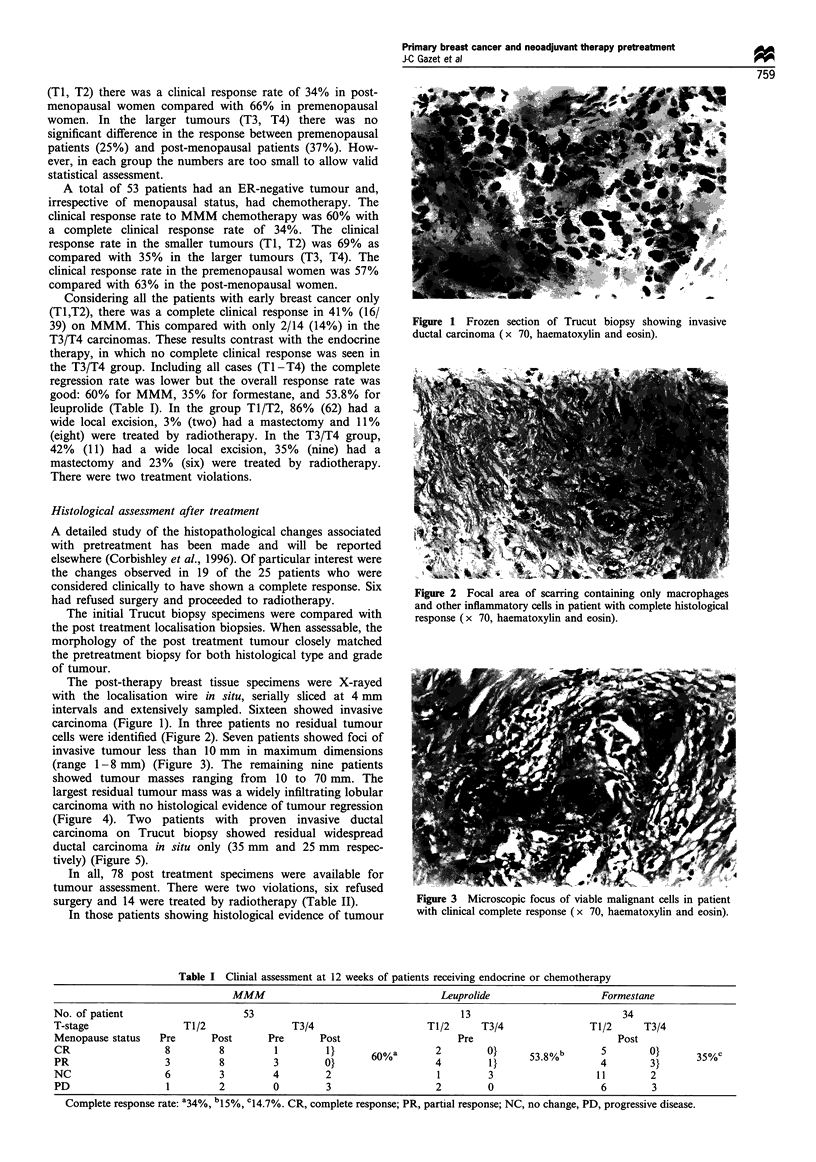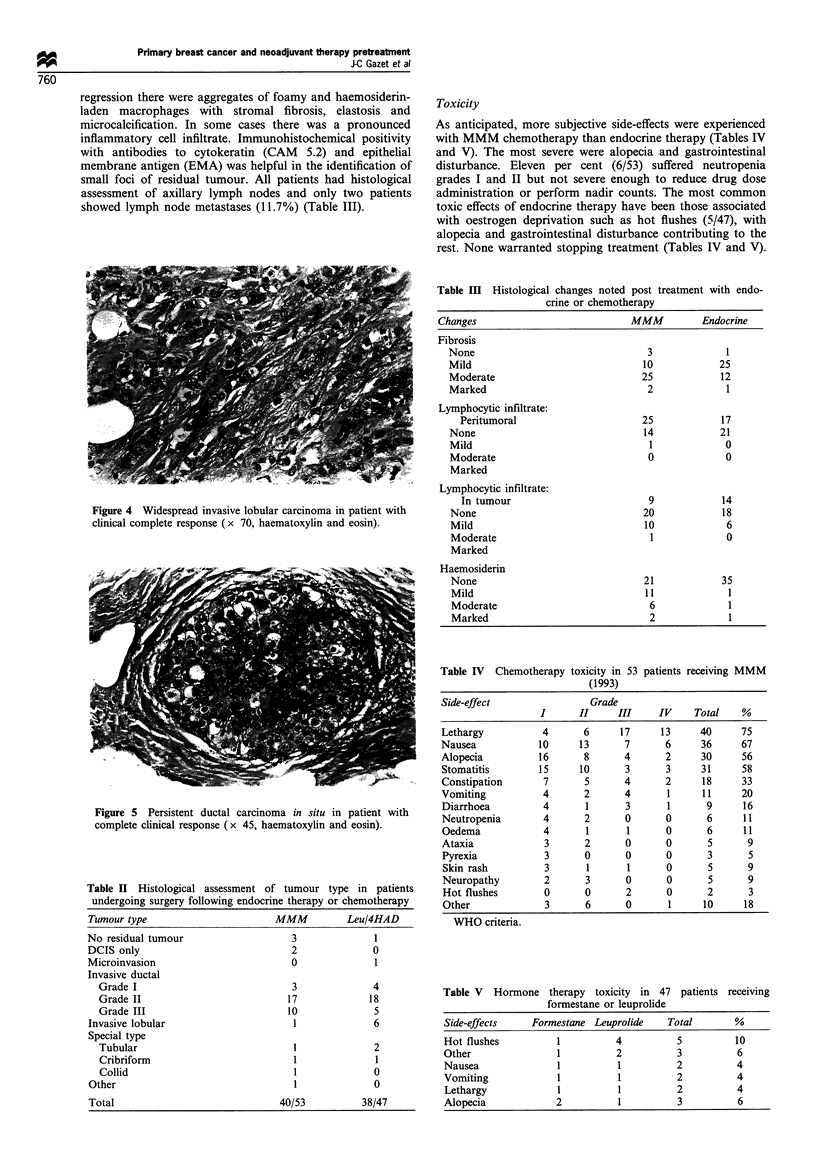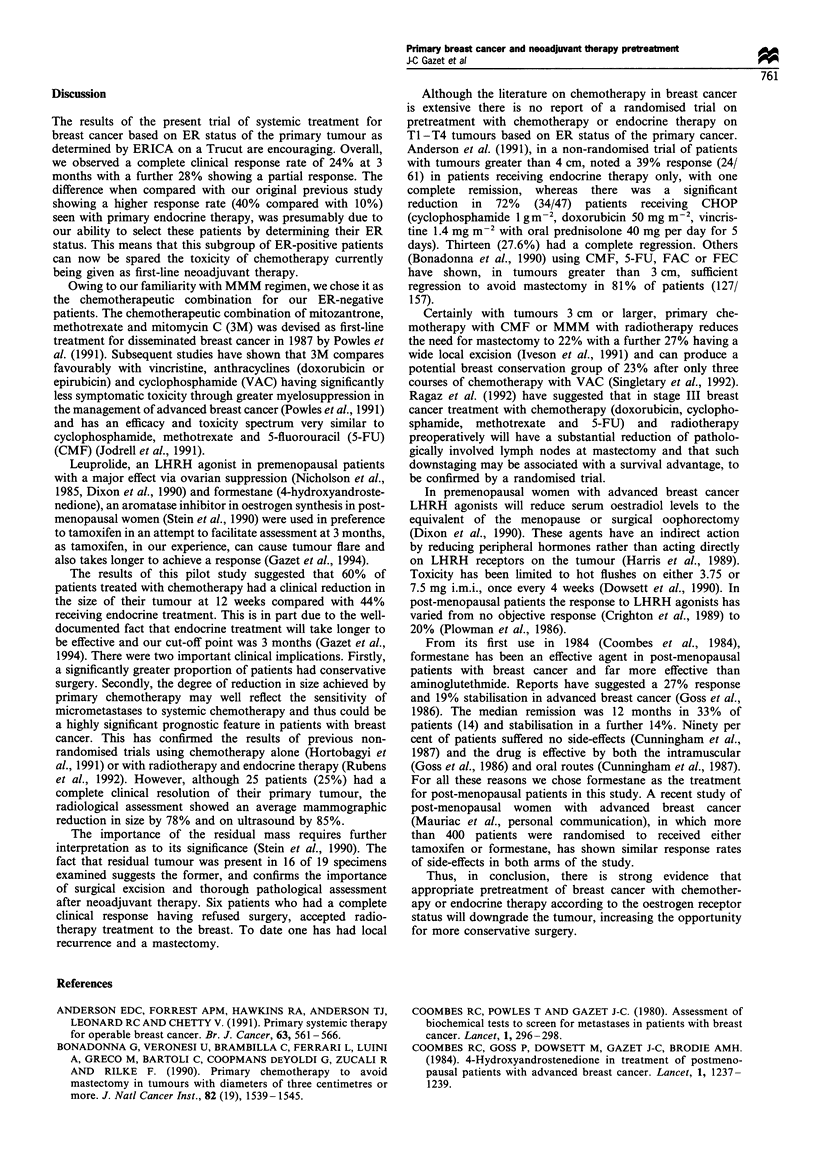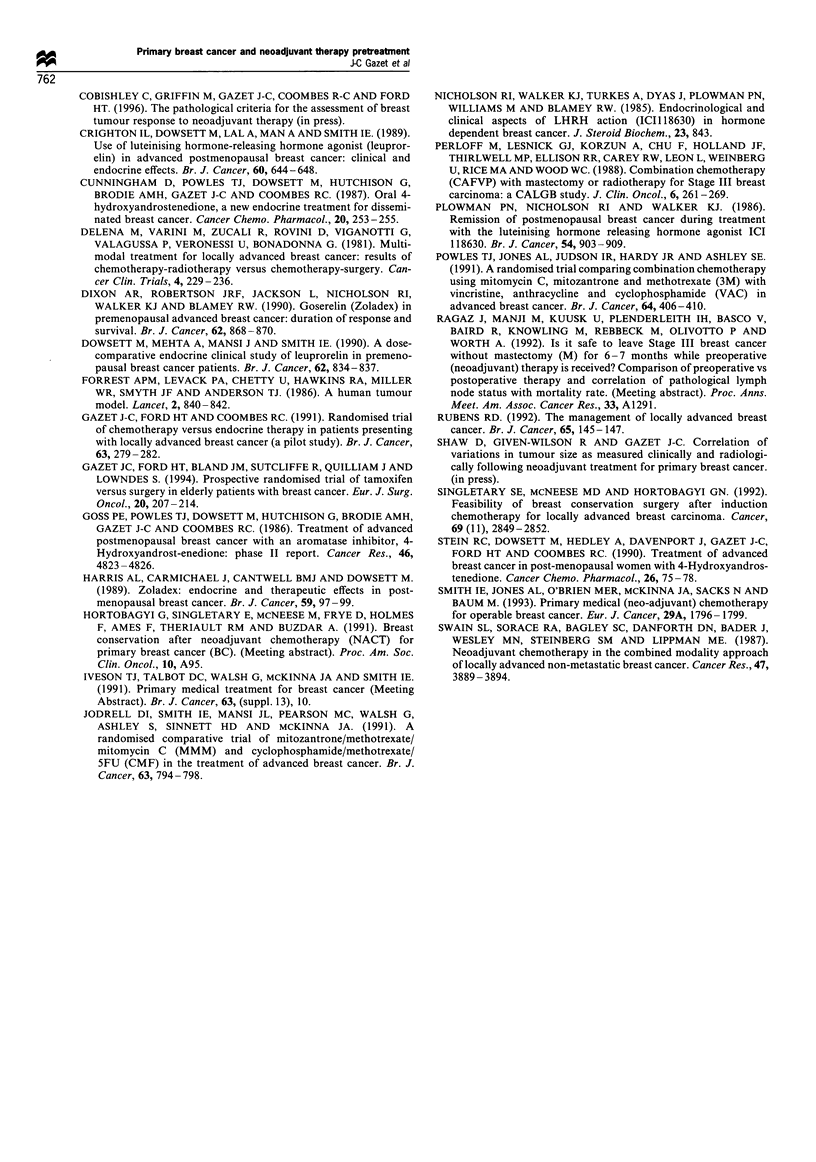# Assesssment of the effect of pretreatment with neoadjuvant therapy on primary breast cancer.

**DOI:** 10.1038/bjc.1996.132

**Published:** 1996-03

**Authors:** J. C. Gazet, R. C. Coombes, H. T. Ford, M. Griffin, C. Corbishley, V. Makinde, S. Lowndes, J. Quilliam, R. Sutcliffe

**Affiliations:** Breast research Unit, St. George's Hospital, London, UK.

## Abstract

**Images:**


British Journal of Cancer (1996) 73, 758-762
© 1996 Stockton Press All rights reserved 0007-0920/96 $12.00

### Assessment of the effect of pretreatment with neoadjuvant therapy on primary breast cancer

J-C Gazet, RC Coombes, HT Ford, M Griffin, C Corbishley, V Makinde, S Lowndes, J Quilliam and R Sutcliffe

*Breast Research Unit, St. George's Hospital, London SW17, UK.*

**Summary** Patients with invasive cancer of the breast (T1-4, N0-2, M0) were assigned to pretreatment based on oestrogen receptor (ER) status; patients with ER-negative tumours received chemotherapy [mitozantrone, methotrexate and mitomycin C (MMM)] for 3 months, patients with ER-positive tumours underwent endocrine therapy [luteinising hormone releasing hormone (LHRH) agonist goserelin (leuprolide–premenopausal) or 4-hydroxyandrostenedione (formestane–post-menopausal)] for 3 months. Of the first 100 patients assessed at 3 months, 47 with ER-positive tumours had a 40.4% response (premenopausal 53.8%; post-menopausal 35%) and 53 with ER-negative tumours had a 60% response (premenopausal 57%; post-menopausal 63%). Patients with early breast cancer (T1/T2) had a complete clinical resolution in 41% (16/39) of cases after MMM and in 20% (7/35) of cases following endocrine therapy compared with 14% (2/14) advanced tumours (T3/T4) following MMM and (0/12) following endocrine therapy. However, in those patients achieving a complete clinical response, subsequent appropriate surgery showed that 16 of 19 patients (84%) had evidence of residual viable tumour on histological examination.

**Keywords:** neoadjuvant therapy; primary breast cancer; pretreatment

Previous studies have demonstrated the excellent reduction in size of primary breast cancer achieved by chemotherapy (Delena *et al.*, 1981; Swain *et al.*, 1987; Perloff *et al.*, 1988) and endocrine therapy (Smith *et al.*, 1993, Forrest *et al.*, 1986).

A randomised trial of primary chemotherapy in patients with advanced primary breast carcinoma has shown that it is more effective in rapidly reducing the size of the primary lesion than endocrine therapy ($P=0.001$) and that it significantly alters the future management of these patients (Gazet *et al.*, 1991). Despite screening, a high proportion of patients still present with T3 or T4 breast carcinoma requiring mastectomy. For this reason we have evaluated the role of neoadjuvant therapy with particular regard to the role of oestrogen receptor (ER) determination by immunocytochemistry using oestrogen receptor immunocytochemical assay (ERICA) as in those patients in which the ER was not evaluated before treatment only 10% responded to endocrine therapy (Gazet *et al.*, 1991).

We hypothesised that endocrine therapy might have a role in neoadjuvant therapy of breast cancer, provided that the ERICA could be performed in the primary tumours, thus indicating which tumours were hormone sensitive.

### Patients, materials and methods

This trial included a series of consecutive patients aged between 30 and 69 (mean 54.02) attending the Combined Breast Clinic at St. George's Hospital between 1990 and 1993 who had a clinical carcinoma of the breast, T1–T4 N0, N1 or N2. This was confirmed by mammographic and fine needle aspiration cytology (FNAC) with no evidence of metastases on standard screening (Coombes *et al.*, 1980), which included chest radiograph, bone scan, liver ultrasound, full blood picture and liver function tests.

All patients were fully informed of the object of the trial. Those joining the trial gave written consent in every case according to the Helsinki agreement. The patients then had a preliminary Trucut biopsy of the tumour to confirm the presence of an invasive ductal or lobular carcinoma. All other malignant tumours including non-invasive carcinoma, ductal breast carcinoma *in situ* (DCIS) and lobular breast carcinoma *in situ* (LCIS), were excluded from the trial.

Those patients who on Trucut biopsy had an ER-positive invasive carcinoma (more than 30% of carcinoma cells staining for ERICA) received endocrine treatment; premenopausal–leuprorelin [a luteinising hormone releasing hormone (LHRH) agonist leuprolide] 3.75 mg subcutaneously every 4 weeks; or post-menopausal–4-hydroxyandrostenedione (formestane), 250 mg i.m. every 2 weeks. Those patients who were ER negative on Trucut biopsy received chemotherapy; MMM–mitozantrone 7 mg $m^{-2}$, every 3 weeks, mitomycin C 7 mg $m^{-2}$ every 6 weeks, methotrexate 30 mg $m^{-2}$ every 3 weeks with folinic acid rescue 15 mg 4–6 hourly, six doses starting 24 h after infusion; four courses in 3 months (12 weeks).

All patients were clinically assessed at 4 weekly intervals. Tumour size was measured with calipers accurately by bidimensional measurements and the area calculated as the sum of these two measurements. All patients had a preliminary mammogram before treatment and a second mammogram 12 weeks before definitive surgery and radiotherapy. A peripheral blood count was performed 21 days before each course of chemotherapy. Nadir counts (day 10–14) were not performed. All responses were assessed according to standard UICC criteria and toxicity according to WHO grading.

At 3 months, following clinical and investigative reassessment, which included mammography and full standard screening, those patients treated by primary chemotherapy or endocrine therapy received appropriate surgery and/or radiotherapy depending on the post-treatment clinical TNM staging.

### Results

Of the first 100 patients assessed at 3 months, 47 had ER-positive tumours and therefore received endocrine therapy according to their menopausal status. The clinical response rate was 53.8% in the premenopausal patients compared with 35% in the post-menopausal patients. In the smaller tumours

Correspondence: J-C Gazet, 104 Harley Street, London W1N 1AF, UK
Received 4 May 1995; revised 10 October 1995; accepted 20 October 1995

(T1, T2) there was a clinical response rate of 34% in post-menopausal women compared with 66% in premenopausal women. In the larger tumours (T3, T4) there was no significant difference in the response between premenopausal patients (25%) and post-menopausal patients (37%). However, in each group the numbers are too small to allow valid statistical assessment.

A total of 53 patients had an ER-negative tumour and, irrespective of menopausal status, had chemotherapy. The clinical response rate to MMM chemotherapy was 60% with a complete clinical response rate of 34%. The clinical response rate in the smaller tumours (T1, T2) was 69% as compared with 35% in the larger tumours (T3, T4). The clinical response rate in the premenopausal women was 57% compared with 63% in the post-menopausal women.

Considering all the patients with early breast cancer only (T1,T2), there was a complete clinical response in 41% (16/39) on MMM. This compared with only 2/14 (14%) in the T3/T4 carcinomas. These results contrast with the endocrine therapy, in which no complete clinical response was seen in the T3/T4 group. Including all cases (T1–T4) the complete regression rate was lower but the overall response rate was good: 60% for MMM, 35% for formestane, and 53.8% for leuprolide (Table I). In the group T1/T2, 86% (62) had a wide local excision, 3% (two) had a mastectomy and 11% (eight) were treated by radiotherapy. In the T3/T4 group, 42% (11) had a wide local excision, 35% (nine) had a mastectomy and 23% (six) were treated by radiotherapy. There were two treatment violations.

### *Histological assessment after treatment*

A detailed study of the histopathological changes associated with pretreatment has been made and will be reported elsewhere (Corbishley *et al.*, 1996). Of particular interest were the changes observed in 19 of the 25 patients who were considered clinically to have shown a complete response. Six had refused surgery and proceeded to radiotherapy.

The initial Trucut biopsy specimens were compared with the post treatment localisation biopsies. When assessable, the morphology of the post treatment tumour closely matched the pretreatment biopsy for both histological type and grade of tumour.

The post-therapy breast tissue specimens were X-rayed with the localisation wire *in situ*, serially sliced at 4 mm intervals and extensively sampled. Sixteen showed invasive carcinoma (Figure 1). In three patients no residual tumour cells were identified (Figure 2). Seven patients showed foci of invasive tumour less than 10 mm in maximum dimensions (range 1–8 mm) (Figure 3). The remaining nine patients showed tumour masses ranging from 10 to 70 mm. The largest residual tumour mass was a widely infiltrating lobular carcinoma with no histological evidence of tumour regression (Figure 4). Two patients with proven invasive ductal carcinoma on Trucut biopsy showed residual widespread ductal carcinoma *in situ* only (35 mm and 25 mm respectively) (Figure 5).

In all, 78 post treatment specimens were available for tumour assessment. There were two violations, six refused surgery and 14 were treated by radiotherapy (Table II).

In those patients showing histological evidence of tumour

**Figure 1** Frozen section of Trucut biopsy showing invasive ductal carcinoma (× 70, haematoxylin and eosin).

**Figure 2** Focal area of scarring containing only macrophages and other inflammatory cells in patient with complete histological response (× 70, haematoxylin and eosin).

**Figure 3** Microscopic focus of viable malignant cells in patient with clinical complete response (× 70, haematoxylin and eosin).

**Table I** Clinial assessment at 12 weeks of patients receiving endocrine or chemotherapy

| | *MMM* | | | | | *Leuprolide* | | | *Formestane* | | |
|---|---|---|---|---|---|---|---|---|---|---|---|
| No. of patient | 53 | | | | | 13 | | | 34 | | |
| T-stage | T1/2 | | T3/4 | | | T1/2 | T3/4 | | T1/2 | T3/4 | |
| Menopause status | Pre | Post | Pre | Post | | Pre | | | Post | | |
| CR | 8 | 8 | 1 | 1} | 60%[a] | 2 | 0} | 53.8%[b] | 5 | 0} | 35%[c] |
| PR | 3 | 8 | 3 | 0} | | 4 | 1} | | 4 | 3} | |
| NC | 6 | 3 | 4 | 2 | | 1 | 3 | | 11 | 2 | |
| PD | 1 | 2 | 0 | 3 | | 2 | 0 | | 6 | 3 | |

Complete response rate: [a]34%, [b]15%, [c]14.7%. CR, complete response; PR, partial response; NC, no change, PD, progressive disease.

regression there were aggregates of foamy and haemosiderin-laden macrophages with stromal fibrosis, elastosis and microcalcification. In some cases there was a pronounced inflammatory cell infiltrate. Immunohistochemical positivity with antibodies to cytokeratin (CAM 5.2) and epithelial membrane antigen (EMA) was helpful in the identification of small foci of residual tumour. All patients had histological assessment of axillary lymph nodes and only two patients showed lymph node metastases (11.7%) (Table III).

**Figure 4** Widespread invasive lobular carcinoma in patient with clinical complete response (× 70, haematoxylin and eosin).

**Figure 5** Persistent ductal carcinoma *in situ* in patient with complete clinical response (× 45, haematoxylin and eosin).

**Table II** Histological assessment of tumour type in patients undergoing surgery following endocrine therapy or chemotherapy

| *Tumour type* | *MMM* | *Leu/4HAD* |
|---|---|---|
| No residual tumour | 3 | 1 |
| DCIS only | 2 | 0 |
| Microinvasion | 0 | 1 |
| Invasive ductal | | |
| Grade I | 3 | 4 |
| Grade II | 17 | 18 |
| Grade III | 10 | 5 |
| Invasive lobular | 1 | 6 |
| Special type | | |
| Tubular | 1 | 2 |
| Cribriform | 1 | 1 |
| Collid | 1 | 0 |
| Other | 1 | 0 |
| Total | 40/53 | 38/47 |

*Toxicity*

As anticipated, more subjective side-effects were experienced with MMM chemotherapy than endocrine therapy (Tables IV and V). The most severe were alopecia and gastrointestinal disturbance. Eleven per cent (6/53) suffered neutropenia grades I and II but not severe enough to reduce drug dose administration or perform nadir counts. The most common toxic effects of endocrine therapy have been those associated with oestrogen deprivation such as hot flushes (5/47), with alopecia and gastrointestinal disturbance contributing to the rest. None warranted stopping treatment (Tables IV and V).

**Table III** Histological changes noted post treatment with endocrine or chemotherapy

| *Changes* | *MMM* | *Endocrine* |
|---|---|---|
| Fibrosis | | |
| None | 3 | 1 |
| Mild | 10 | 25 |
| Moderate | 25 | 12 |
| Marked | 2 | 1 |
| Lymphocytic infiltrate: | | |
| Peritumoral | 25 | 17 |
| None | 14 | 21 |
| Mild | 1 | 0 |
| Moderate | 0 | 0 |
| Marked | | |
| Lymphocytic infiltrate: | | |
| In tumour | 9 | 14 |
| None | 20 | 18 |
| Mild | 10 | 6 |
| Moderate | 1 | 0 |
| Marked | | |
| Haemosiderin | | |
| None | 21 | 35 |
| Mild | 11 | 1 |
| Moderate | 6 | 1 |
| Marked | 2 | 1 |

**Table IV** Chemotherapy toxicity in 53 patients receiving MMM (1993)

| *Side-effect* | *Grade* | | | | | |
|---|---|---|---|---|---|---|
| | *I* | *II* | *III* | *IV* | *Total* | *%* |
| Lethargy | 4 | 6 | 17 | 13 | 40 | 75 |
| Nausea | 10 | 13 | 7 | 6 | 36 | 67 |
| Alopecia | 16 | 8 | 4 | 2 | 30 | 56 |
| Stomatitis | 15 | 10 | 3 | 3 | 31 | 58 |
| Constipation | 7 | 5 | 4 | 2 | 18 | 33 |
| Vomiting | 4 | 2 | 4 | 1 | 11 | 20 |
| Diarrhoea | 4 | 1 | 3 | 1 | 9 | 16 |
| Neutropenia | 4 | 2 | 0 | 0 | 6 | 11 |
| Oedema | 4 | 1 | 1 | 0 | 6 | 11 |
| Ataxia | 3 | 2 | 0 | 0 | 5 | 9 |
| Pyrexia | 3 | 0 | 0 | 0 | 3 | 5 |
| Skin rash | 3 | 1 | 1 | 0 | 5 | 9 |
| Neuropathy | 2 | 3 | 0 | 0 | 5 | 9 |
| Hot flushes | 0 | 0 | 2 | 0 | 2 | 3 |
| Other | 3 | 6 | 0 | 1 | 10 | 18 |

WHO criteria.

**Table V** Hormone therapy toxicity in 47 patients receiving formestane or leuprolide

| *Side-effects* | *Formestane* | *Leuprolide* | *Total* | *%* |
|---|---|---|---|---|
| Hot flushes | 1 | 4 | 5 | 10 |
| Other | 1 | 2 | 3 | 6 |
| Nausea | 1 | 1 | 2 | 4 |
| Vomiting | 1 | 1 | 2 | 4 |
| Lethargy | 1 | 1 | 2 | 4 |
| Alopecia | 2 | 1 | 3 | 6 |

### Discussion

The results of the present trial of systemic treatment for breast cancer based on ER status of the primary tumour as determined by ERICA on a Trucut are encouraging. Overall, we observed a complete clinical response rate of 24% at 3 months with a further 28% showing a partial response. The difference when compared with our original previous study showing a higher response rate (40% compared with 10%) seen with primary endocrine therapy, was presumably due to our ability to select these patients by determining their ER status. This means that this subgroup of ER-positive patients can now be spared the toxicity of chemotherapy currently being given as first-line neoadjuvant therapy.

Owing to our familiarity with MMM regimen, we chose it as the chemotherapeutic combination for our ER-negative patients. The chemotherapeutic combination of mitozantrone, methotrexate and mitomycin C (3M) was devised as first-line treatment for disseminated breast cancer in 1987 by Powles *et al.* (1991). Subsequent studies have shown that 3M compares favourably with vincristine, anthracyclines (doxorubicin or epirubicin) and cyclophosphamide (VAC) having significantly less symptomatic toxicity through greater myelosuppression in the management of advanced breast cancer (Powles *et al.*, 1991) and has an efficacy and toxicity spectrum very similar to cyclophosphamide, methotrexate and 5-fluorouracil (5-FU) (CMF) (Jodrell *et al.*, 1991).

Leuprolide, an LHRH agonist in premenopausal patients with a major effect via ovarian suppression (Nicholson *et al.*, 1985, Dixon *et al.*, 1990) and formestane (4-hydroxyandrostenedione), an aromatase inhibitor in oestrogen synthesis in post-menopausal women (Stein *et al.*, 1990) were used in preference to tamoxifen in an attempt to facilitate assessment at 3 months, as tamoxifen, in our experience, can cause tumour flare and also takes longer to achieve a response (Gazet *et al.*, 1994).

The results of this pilot study suggested that 60% of patients treated with chemotherapy had a clinical reduction in the size of their tumour at 12 weeks compared with 44% receiving endocrine treatment. This is in part due to the well-documented fact that endocrine treatment will take longer to be effective and our cut-off point was 3 months (Gazet *et al.*, 1994). There were two important clinical implications. Firstly, a significantly greater proportion of patients had conservative surgery. Secondly, the degree of reduction in size achieved by primary chemotherapy may well reflect the sensitivity of micrometastases to systemic chemotherapy and thus could be a highly significant prognostic feature in patients with breast cancer. This has confirmed the results of previous non-randomised trials using chemotherapy alone (Hortobagyi *et al.*, 1991) or with radiotherapy and endocrine therapy (Rubens *et al.*, 1992). However, although 25 patients (25%) had a complete clinical resolution of their primary tumour, the radiological assessment showed an average mammographic reduction in size by 78% and on ultrasound by 85%.

The importance of the residual mass requires further interpretation as to its significance (Stein *et al.*, 1990). The fact that residual tumour was present in 16 of 19 specimens examined suggests the former, and confirms the importance of surgical excision and thorough pathological assessment after neoadjuvant therapy. Six patients who had a complete clinical response having refused surgery, accepted radiotherapy treatment to the breast. To date one has had local recurrence and a mastectomy.

Although the literature on chemotherapy in breast cancer is extensive there is no report of a randomised trial on pretreatment with chemotherapy or endocrine therapy on T1–T4 tumours based on ER status of the primary cancer. Anderson *et al.* (1991), in a non-randomised trial of patients with tumours greater than 4 cm, noted a 39% response (24/61) in patients receiving endocrine therapy only, with one complete remission, whereas there was a significant reduction in 72% (34/47) patients receiving CHOP (cyclophosphamide 1 g m$^{-2}$, doxorubicin 50 mg m$^{-2}$, vincristine 1.4 mg m$^{-2}$ with oral prednisolone 40 mg per day for 5 days). Thirteen (27.6%) had a complete regression. Others (Bonadonna *et al.*, 1990) using CMF, 5-FU, FAC or FEC have shown, in tumours greater than 3 cm, sufficient regression to avoid mastectomy in 81% of patients (127/157).

Certainly with tumours 3 cm or larger, primary chemotherapy with CMF or MMM with radiotherapy reduces the need for mastectomy to 22% with a further 27% having a wide local excision (Iveson *et al.*, 1991) and can produce a potential breast conservation group of 23% after only three courses of chemotherapy with VAC (Singletary *et al.*, 1992). Ragaz *et al.* (1992) have suggested that in stage III breast cancer treatment with chemotherapy (doxorubicin, cyclophosphamide, methotrexate and 5-FU) and radiotherapy preoperatively will have a substantial reduction of pathologically involved lymph nodes at mastectomy and that such downstaging may be associated with a survival advantage, to be confirmed by a randomised trial.

In premenopausal women with advanced breast cancer LHRH agonists will reduce serum oestradiol levels to the equivalent of the menopause or surgical oophorectomy (Dixon *et al.*, 1990). These agents have an indirect action by reducing peripheral hormones rather than acting directly on LHRH receptors on the tumour (Harris *et al.*, 1989). Toxicity has been limited to hot flushes on either 3.75 or 7.5 mg i.m.i., once every 4 weeks (Dowsett *et al.*, 1990). In post-menopausal patients the response to LHRH agonists has varied from no objective response (Crighton *et al.*, 1989) to 20% (Plowman *et al.*, 1986).

From its first use in 1984 (Coombes *et al.*, 1984), formestane has been an effective agent in post-menopausal patients with breast cancer and far more effective than aminoglutethmide. Reports have suggested a 27% response and 19% stabilisation in advanced breast cancer (Goss *et al.*, 1986). The median remission was 12 months in 33% of patients (14) and stabilisation in a further 14%. Ninety per cent of patients suffered no side-effects (Cunningham *et al.*, 1987) and the drug is effective by both the intramuscular (Goss *et al.*, 1986) and oral routes (Cunningham *et al.*, 1987). For all these reasons we chose formestane as the treatment for post-menopausal patients in this study. A recent study of post-menopausal women with advanced breast cancer (Mauriac *et al.*, personal communication), in which more than 400 patients were randomised to received either tamoxifen or formestane, has shown similar response rates of side-effects in both arms of the study.

Thus, in conclusion, there is strong evidence that appropriate pretreatment of breast cancer with chemotherapy or endocrine therapy according to the oestrogen receptor status will downgrade the tumour, increasing the opportunity for more conservative surgery.

### References

ANDERSON EDC, FORREST APM, HAWKINS RA, ANDERSON TJ, LEONARD RC AND CHETTY V. (1991). Primary systemic therapy for operable breast cancer. *Br. J. Cancer*, **63**, 561–566.

BONADONNA G, VERONESI U, BRAMBILLA C, FERRARI L, LUINI A, GRECO M, BARTOLI C, COOPMANS DEYOLDI G, ZUCALI R AND RILKE F. (1990). Primary chemotherapy to avoid mastectomy in tumours with diameters of three centimetres or more. *J. Natl Cancer Inst.*, **82** (19), 1539–1545.

COOMBES RC, POWLES T AND GAZET J-C. (1980). Assessment of biochemical tests to screen for metastases in patients with breast cancer. *Lancet*, **1**, 296–298.

COOMBES RC, GOSS P, DOWSETT M, GAZET J-C, BRODIE AMH. (1984). 4-Hydroxyandrostenedione in treatment of postmenopausal patients with advanced breast cancer. *Lancet*, **1**, 1237–1239.

COBISHLEY C, GRIFFIN M, GAZET J-C, COOMBES R-C AND FORD HT. (1996). The pathological criteria for the assessment of breast tumour response to neoadjuvant therapy (in press).

CRIGHTON IL, DOWSETT M, LAL A, MAN A AND SMITH IE. (1989). Use of luteinising hormone-releasing hormone agonist (leuprorelin) in advanced postmenopausal breast cancer: clinical and endocrine effects. *Br. J. Cancer*, **60**, 644–648.

CUNNINGHAM D, POWLES TJ, DOWSETT M, HUTCHISON G, BRODIE AMH, GAZET J-C AND COOMBES RC. (1987). Oral 4-hydroxyandrostenedione, a new endocrine treatment for disseminated breast cancer. *Cancer Chemo. Pharmacol.*, **20**, 253–255.

DELENA M, VARINI M, ZUCALI R, ROVINI D, VIGANOTTI G, VALAGUSSA P, VERONESSI U, BONADONNA G. (1981). Multimodal treatment for locally advanced breast cancer: results of chemotherapy-radiotherapy versus chemotherapy-surgery. *Cancer Clin. Trials*, **4**, 229–236.

DIXON AR, ROBERTSON JRF, JACKSON L, NICHOLSON RI, WALKER KJ AND BLAMEY RW. (1990). Goserelin (Zoladex) in premenopausal advanced breast cancer: duration of response and survival. *Br. J. Cancer*, **62**, 868–870.

DOWSETT M, MEHTA A, MANSI J AND SMITH IE. (1990). A dose-comparative endocrine clinical study of leuprorelin in premenopausal breast cancer patients. *Br. J. Cancer*, **62**, 834–837.

FORREST APM, LEVACK PA, CHETTY U, HAWKINS RA, MILLER WR, SMYTH JF AND ANDERSON TJ. (1986). A human tumour model. *Lancet*, **2**, 840–842.

GAZET J-C, FORD HT AND COOMBES RC. (1991). Randomised trial of chemotherapy versus endocrine therapy in patients presenting with locally advanced breast cancer (a pilot study). *Br. J. Cancer*, **63**, 279–282.

GAZET JC, FORD HT, BLAND JM, SUTCLIFFE R, QUILLIAM J AND LOWNDES S. (1994). Prospective randomised trial of tamoxifen versus surgery in elderly patients with breast cancer. *Eur. J. Surg. Oncol.*, **20**, 207–214.

GOSS PE, POWLES TJ, DOWSETT M, HUTCHISON G, BRODIE AMH, GAZET J-C AND COOMBES RC. (1986). Treatment of advanced postmenopausal breast cancer with an aromatase inhibitor, 4-Hydroxyandrost-enedione: phase II report. *Cancer Res.*, **46**, 4823–4826.

HARRIS AL, CARMICHAEL J, CANTWELL BMJ AND DOWSETT M. (1989). Zoladex: endocrine and therapeutic effects in post-menopausal breast cancer. *Br. J. Cancer*, **59**, 97–99.

HORTOBAGYI G, SINGLETARY E, MCNEESE M, FRYE D, HOLMES F, AMES F, THERIAULT RM AND BUZDAR A. (1991). Breast conservation after neoadjuvant chemotherapy (NACT) for primary breast cancer (BC). (Meeting abstract). *Proc. Am. Soc. Clin. Oncol.*, **10**, A95.

IVESON TJ, TALBOT DC, WALSH G, MCKINNA JA AND SMITH IE. (1991). Primary medical treatment for breast cancer (Meeting Abstract). *Br. J. Cancer*, **63**, (suppl. 13), 10.

JODRELL DI, SMITH IE, MANSI JL, PEARSON MC, WALSH G, ASHLEY S, SINNETT HD AND MCKINNA JA. (1991). A randomised comparative trial of mitozantrone/methotrexate/ mitomycin C (MMM) and cyclophosphamide/methotrexate/ 5FU (CMF) in the treatment of advanced breast cancer. *Br. J. Cancer*, **63**, 794–798.

NICHOLSON RI, WALKER KJ, TURKES A, DYAS J, PLOWMAN PN, WILLIAMS M AND BLAMEY RW. (1985). Endocrinological and clinical aspects of LHRH action (ICI118630) in hormone dependent breast cancer. *J. Steroid Biochem.*, **23**, 843.

PERLOFF M, LESNICK GJ, KORZUN A, CHU F, HOLLAND JF, THIRLWELL MP, ELLISON RR, CAREY RW, LEON L, WEINBERG U, RICE MA AND WOOD WC. (1988). Combination chemotherapy (CAFVP) with mastectomy or radiotherapy for Stage III breast carcinoma: a CALGB study. *J. Clin. Oncol.*, **6**, 261–269.

PLOWMAN PN, NICHOLSON RI AND WALKER KJ. (1986). Remission of postmenopausal breast cancer during treatment with the luteinising hormone releasing hormone agonist ICI 118630. *Br. J. Cancer*, **54**, 903–909.

POWLES TJ, JONES AL, JUDSON IR, HARDY JR AND ASHLEY SE. (1991). A randomised trial comparing combination chemotherapy using mitomycin C, mitozantrone and methotrexate (3M) with vincristine, anthracycline and cyclophosphamide (VAC) in advanced breast cancer. *Br. J. Cancer*, **64**, 406–410.

RAGAZ J, MANJI M, KUUSK U, PLENDERLEITH IH, BASCO V, BAIRD R, KNOWLING M, REBBECK M, OLIVOTTO P AND WORTH A. (1992). Is it safe to leave Stage III breast cancer without mastectomy (M) for 6–7 months while preoperative (neoadjuvant) therapy is received? Comparison of preoperative vs postoperative therapy and correlation of pathological lymph node status with mortality rate. (Meeting abstract). *Proc. Anns. Meet. Am. Assoc. Cancer Res.*, **33**, A1291.

RUBENS RD. (1992). The management of locally advanced breast cancer. *Br. J. Cancer*, **65**, 145–147.

SHAW D, GIVEN-WILSON R AND GAZET J-C. Correlation of variations in tumour size as measured clinically and radiologically following neoadjuvant treatment for primary breast cancer. (in press).

SINGLETARY SE, MCNEESE MD AND HORTOBAGYI GN. (1992). Feasibility of breast conservation surgery after induction chemotherapy for locally advanced breast carcinoma. *Cancer*, **69** (11), 2849–2852.

STEIN RC, DOWSETT M, HEDLEY A, DAVENPORT J, GAZET J-C, FORD HT AND COOMBES RC. (1990). Treatment of advanced breast cancer in post-menopausal women with 4-Hydroxyandrostenedione. *Cancer Chemo. Pharmacol.*, **26**, 75–78.

SMITH IE, JONES AL, O'BRIEN MER, MCKINNA JA, SACKS N AND BAUM M. (1993). Primary medical (neo-adjuvant) chemotherapy for operable breast cancer. *Eur. J. Cancer*, **29A**, 1796–1799.

SWAIN SL, SORACE RA, BAGLEY SC, DANFORTH DN, BADER J, WESLEY MN, STEINBERG SM AND LIPPMAN ME. (1987). Neoadjuvant chemotherapy in the combined modality approach of locally advanced non-metastatic breast cancer. *Cancer Res.*, **47**, 3889–3894.